# In Vivo Thermal Ablation of Deep Intrahepatic Targets Using a Super-Convergent MRgHIFU Applicator and a Pseudo-Tumor Model

**DOI:** 10.3390/cancers15153961

**Published:** 2023-08-03

**Authors:** Orane Lorton, Pauline Coralie Guillemin, Andrea Peloso, Yacine M’Rad, Lindsey Alexandra Crowe, Thibaud Koessler, Pierre-Alexandre Poletti, Sana Boudabbous, Alexis Ricoeur, Rares Salomir

**Affiliations:** 1 Image Guided Interventions Laboratory (GR-949), Faculty of Medicine, University of Geneva, 1211 Geneva, Switzerland; 2 Visceral Surgery Division, University Hospitals of Geneva, 1205 Geneva, Switzerland; 3 Radiology Division, University Hospitals of Geneva, 1205 Geneva, Switzerland; 4 Oncology Department, University Hospitals of Geneva, 1205 Geneva, Switzerland

**Keywords:** high-intensity focused ultrasound, thermal ablation, liver ablation, hyperthermia, targeting, tumor-mimicking RF markers

## Abstract

**Simple Summary:**

Magnetic-resonance-guided high-intensity focused ultrasound (MRgHIFU) is a promising technology for ablation of liver tumors not eligible for surgery. This study aimed to demonstrate the workflow feasibility and the spatial accuracy of a novel concept of MRgHIFU transducer dedicated to deep intrahepatic targets on six in vivo pig livers. Before the MRgHIFU ablation, a histological marker mimicking a metastasis was defined in a region considered as challenging to resect. The pseudo-tumor, visible on pre-operative MR images and post-mortem gross pathology, was the target for validation of the entire workflow and the relevancy of the novel concept of HIFU transducer. The presence of the MRgHIFU ablations on gross pathology at the expected locations confirmed the ability to induce transcostal thermal lesions by MRgHIFU in challenging tumors. No relevant side effects on near-field tissues such as the skin or ribs were evidenced.

**Abstract:**

Background: HIFU ablation of liver malignancies is particularly challenging due to respiratory motion, high tissue perfusion and the presence of the rib cage. Based on our previous development of a super-convergent phased-array transducer, we aimed to further investigate, in vivo, its applicability to deep intrahepatic targets. Methods: In a series of six pigs, a pseudo-tumor model was used as target, visible both on intra-operatory MRI and post-mortem gross pathology. The transcostal MRgHIFU ablation was prescribed coplanar with the pseudo-tumor, either axial or sagittal, but deliberately shifted 7 to 18 mm to the side. No specific means of protection of the ribs were implemented. Post-treatment MRI follow-up was performed at D7, followed by animal necropsy and gross pathology of the liver. Results: The pseudo-tumor was clearly identified on T1w MR imaging and subsequently allowed the MRgHIFU planning. The peak temperature at the focal point ranged from 58–87 °C. Gross pathology confirmed the presence of the pseudo-tumor and the well-delineated MRgHIFU ablation at the expected locations. Conclusions: The specific design of the transducer enabled a reliable workflow. It demonstrated a good safety profile for in vivo transcostal MRgHIFU ablation of deep-liver targets, graded as challenging for standard surgery.

## 1. Introduction

Liver cancer is the sixth most common cancer worldwide, with more than 900,000 new liver cancer cases in 2020, and the second leading cause of cancer-related death, with more than 830,000 deaths [1,2,3]. Surgery remains the gold standard for the treatment of hepatocellular carcinoma (HCC) [4,5]; however, less than 25% [6,7] of the patients are eligible for liver resection. The resectability criteria take into consideration the number of lesions; the tumor location, particularly the proximity to blood vessels and residual perfusion; and the tumor size and margins to ensure a curative treatment and a viable volume of healthy tissue left after resection [4,5,8,9,10,11,12]. Local ablation is routinely performed on small tumors [13] (≤3 cm) and in patients non-amenable to surgery. The tumor macro- and micro-environment is a complex physical medium to be considered when planning successful thermal therapy [12,14]. The two main techniques used in clinical routine to induce tissue necrosis by heating are microwave (MW) and radiofrequency (RF) ablations [15,16,17,18,19,20]. Both are based on the local generation of electromagnetic waves but working at different frequencies, 400–500 kHz for the RF and 900–2500 MHz for the MW. The benefits of these techniques are similar in terms of overall survival, complete ablation, recurrence-free survival and local tumor progression [21,22,23,24,25,26]. Potential complications such as bleeding, colon perforation, bile duct stenosis or hemothorax [16,27] are also the same. However, MW ablation induces more homogeneous thermal ablation than RF, and the faster heating process compared with RF ablation leads to less sensitivity to the heat-sink effect. The technology of laser ablation also demonstrated a reliable outcome on patients suffering from hepato-cellular carcinoma [28] but the required equipment is more sophisticated [17]. Conversely, cryoablation uses extremely low temperatures to induce freezing and thawing, causing several types of cell damage, such as cell dehydration or membrane disruption leading to cell death [17,29,30,31]. The cryotherapy can be monitored under computed tomography (CT), ultrasound (US) or magnetic resonance imaging (MRI), and large tumors can be treated by using multiple probes simultaneously. Rong et al. [32] demonstrated a complete response rate of 99.4% for tumors <3 cm over 1595 patients, but bleedings in cryoablation can be more serious than in thermal ablations due to the absence of coagulation or cauterisation. The inflammatory response can also induce severe injuries, named cryoshock, leading to hypotension, multi-organ or respiratory failures [33]. Nanoparticle-induced thermal ablation has also been investigated for the capacity of nanoparticles to absorb the light, electromagnetic or RF waves and their ability to improve the heating in tumors [34]. These include magnetic [35] or gold nanoparticles [36]. Nanoparticles could specifically target the cancer cells and induce thermal lesions in the tumor while avoiding damage in surrounding tissues. However, to ensure a homogeneous distribution of nanoparticles inside the tumor is challenging, and the nanoparticles may remain in the tissues for months, depending on the nature of the nanoparticles.

Another modality for thermal ablation is high-intensity focused ultrasound (HIFU) [37]. Compared with the others, HIFU has the advantage to be non-invasive and can be monitored by magnetic resonance imaging (MRgHIFU), allowing 3D targeting and near real-time MR thermometry [38,39,40]. Temperature elevation maps are generated based on the proton resonance frequency shift (PRFS) method and ensure a controlled lesion while minimizing the risks of thermal injuries in surrounding tissues [41,42].

HIFU ablation of hepatic tissues is challenging due to respiratory motion, the presence of the rib cage and the proximity and risk to organs or major blood vessels [43,44,45,46,47]. Some techniques have been developed to solve the motion issue, such as applying sonications during the quasi-stationary period of the breathing cycle, namely gating, or using motion-compensation algorithms based on motion tracking to adjust the emitted power, the target or the position of the transducer [44,47,48,49,50,51,52,53,54,55,56,57,58]. The rib cage is a major issue as it absorbs the acoustic beams and may inhibit the creation of a lesion, while increasing the risk of burns [51,59,60]. Several techniques have been developed to overcome the rib cage issues, such as the partial rib resection [61] or the use of reflective patches located on the skin along the beam pathway [62]. Quesson et al. [63] reported a method consisting in deactivating the acoustic emitters located in front of the ribs by finding the target, manually segmenting the ribs on MR images, and projecting the segmented area on the transducer surface following the beam path. Another approach to avoid rib burn is the adaptive design of the transducer. A linearly segmented transducer populated with 10 emitters has been tested [64] to evaluate the alteration on the pre-focal and focal shape, as well as the width reduction of the HIFU beam when the elements in front of the ribs are switched off. Ramaekers et al. [65] designed a Voronoi-tessellated transducer based on Fermat’s spiral, developed to limit the energy absorption by the ribs based on the elements’ geometry.

Recently, a novel concept of super-convergent transducer dedicated to transcostal hepatic ablation was developed and tested in vivo on two pig livers [66]. The wide aperture, natural deep focusing, and optimization of the active surface with irregular hexagons allowed thermal ablations in deep-liver tissues without the need for rib protection. The performances met expectations, and liver ablations were confirmed 7 days after the intervention by post-mortem evaluation. Before designing clinical studies and applying for regulatory approval, the investigational device requires more consistent evidence in vivo, in terms of safety and spatial accuracy of targeting. Of particular interest are the deep intrahepatic targets graded as inoperable or challenging for surgical resection.

Here, we addressed the in vivo feasibility and spatial accuracy of the device in pig liver, targeting challenging regions using that super-convergent MRgHIFU transducer. A histologic marker, mimicking tumoral metastases, was imprinted, by transcutaneous intervention, in the liver under ultrasound (US) guidance, according to Petrusca et al. [67]. The objectives of the present study were (1) to evaluate the ability of the device to create a HIFU ablation in a defined deep region of the liver considered as challenging to resect, and (2) to validate the whole treatment workflow and to obtain further insights on the near-field safety.

## 2. Materials and Methods

### 2.1. Protocol for In Vivo Experiment

Six female pigs (average weight = 35.6 kg ± 1.5) were sonicated in this study according to the ethical approval from the local animal research committee. As described in Lorton et al. [66], each animal was premedicated 30 min before anesthesia by intramuscular injection of midazolam (0.75 mg/kg). A catheter was placed in an auricular vein and tracheal intubation was carried out. Anesthesia was maintained by a 2% isoflurane (Abbott AG, Baar, Switzerland) air mixture, and the vital parameters were monitored. A Temgesic skin patch (0.01 mg/kg) (Grunenthal, Aix-La-Chapelle, Aachen, Germany), effective for 48 h, was applied prior to animal’s awakening. The animal was positioned prone and the HIFU transducer was maintained under the pig in front of the liver. This setup was preferred with respect to the MR signal stability in PRFS MR thermometry and the available acoustic window for sonicating the liver. Before each MRgHIFU sonication and RF ablation, an intravenous injection of atracurium (0.6 mg/kg) was delivered to prevent any accidental muscle contraction and ensure a stable apnea. The same general anesthesia protocol was performed at 7 days post-surgery (D7) for semi-chronic imaging control. An intravenous injection of Gadolinium (Gd) (0.3 mL/kg) was administered for contrast-enhanced MRI follow-up. The pig was sacrificed by intravenous KCl at lethal dose.

**Figure 1:**
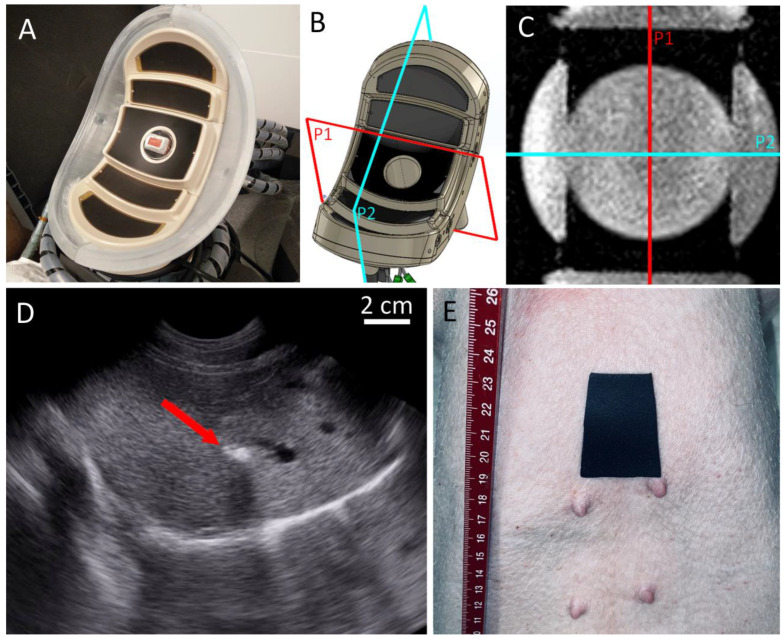
(**A**) Picture of the novel concept of the HIFU transducer, embedding 5 concentric segments, dedicated to liver ablation. A confocal MR-compatible ultrasound imaging probe is available for further use. (**B**) computer-assisted design of the transducer pointing out the two-mirror symmetry orthogonal planes P1 and P2. (**C**) MRI slicing parallel to the transducer, intersecting the three first segments and illustrating the P1 and P2 planes. (**D**) US image of the liver, at the endpoint of the RF ablation that fixes the histological marker. The red arrow indicates the “white cavitation” cloud. (**E**) Ultrasound reflective patch on the pig skin in front of the sternum.

Before HIFU ablations, a radiofrequency (RF) marker mimicking a target metastasis was created in the liver parenchyma under ultrasound guidance to help validate the targeting accuracy achieved by focused ultrasound. The marker was created in a significant location, usually considered difficult to manage by common surgical procedures. The location was chosen in real time by the interventional radiologist, with a depth ranging from 4 to 6 cm, as measured from the anterior capsule of the liver and within anatomic proximity of major hepatic vessels, the biliary duct or other organs (stomach or heart, for instance). Based on the Petrusca et al. study [67], the tumor-mimicking marker consisted in a primary injection of a mixture of contrast agents, an RF ablation for fixation in the tissues, followed by another contrast agent injection. The mixture of 1.5% gadolinium 0.5M (Dotarem, Guerbet, France) for MR contrast was prepared with sterile methylene blue solution (ProveDye, ProvePharm, Marseille, France) for visual assessment of the target during post mortem examination. A volume of 0.1 mL of the solution was injected in the hepatic parenchyma in a suitable location in terms of relevant anatomy and transducer capacities. The RF ablation was then performed using a home-made monopolar RF electrode to fixate the marker by tissue coagulation. The dedicated RF electrode was composed of a sterile 16 G needle of 15 cm length for injection, surrounded by a syringe which was loaded with the mixture. The needle was covered by a polytetrafluoroethylene (PTFE) sheath for electric insulation of the tissues on the path, except the tip for electrical contact. A cut in the sheath was performed at the proximal base of the needle and a conductive clamp was added for electrical conduction from the RF clinical generator to the end of the tip. The electrode was powered by a clinical generator (CELON power System, Olympus, Tokyo, Japan) via a conductive clamp at the beginningof the needle and was grounded using a 6 cm^2^ copper piece laying on the pig’s skin. The injections followed by RF ablations were all performed by the same experienced interventional radiologist under ultrasound (US) guidance using a Chison Sonobook 6 US scanner equipped with an abdominal P2-V probe (CHISON Medical Technologies, Wuxi, China). The phased-array imaging probe was used in harmonic mode at 6.4 MHz. A hyperechoic image was visible on US at the end of the RF ablation due to “white cavitation” bubbles, as illustrated in Figure 1D. This phenomenon served as an indicator of a suitable RF marker and to end the RF procedure. The RF power began at 5 W and was gradually increased until either a “white cavitation” bubble cloud was visible on US images, demonstrating permanent lesioning, or the generator stopped the ablation due to impedance cut-off. The RF ablation was followed by another 0.1 mL injection of the same solution to emphasize the MR and histological contrast agent. After the RF marking, the RF needle was retracted and the skin was locally disinfected. A single puncture was required for the whole procedure, no bleeding was noted, and the time for this entire process did not exceed 20 min.

### 2.2. MR-Guided HIFU

An acoustic reflective patch was placed on the animal’s skin in front of the sternum to avoid burn risks (see Figure 1E). The location of the patch was determined by palpation and using anatomical MR imaging if needed.

Therapeutic ultrasound was generated by a novel concept of phased-array transducer dedicated to transcostal ultrasound ablation of liver tumors as described by Lorton et al. [66] (see Figure 1A). The transducer is composed of 256 hexagonal elements of different sizes and distributed across 5 concentric spherical segments. The element matrix was calculated to optimally populate the transducer surface while avoiding symmetries or redundancies that could lead to secondary lobes. The even and wide energy distribution established by the large active surface minimizes thermal heating in the near field. The large bend radius enables abdominal fitting, and deep focusing naturally focusses at a 10 cm depth. After extensive 3D numerical simulations, and trade-offs between the penetration depth of the ultrasound, the volume reachable by electronic steering, the size of the elements, the impedance matching, and the decoupling from the MR scanner to avoid RF interferences on MR images, the transducer frequency was set at 650 kHz. It was powered by a 256-channel beam former (Image Guided Therapy, Pessac, France) and each element was individually controllable. The acoustic coupling and the active cooling of the transducer were managed by placing a biocompatible membrane on the front side of the transducer. A pump circulated 0.4 L/min of deionized and degassed water, cooled at 17 °C by an MR compatible heat exchanger. The membrane compartment was filled per need using a three-way valve. The tunable volume of water in front of the transducer allowed adjustment of the focal point depth and served to limit the thermal risk to the ribs. The closer the transducer to the skin, the lower the local acoustic intensity on the ribs, and, therefore, the lower the risk of heating.

**Figure 2:**
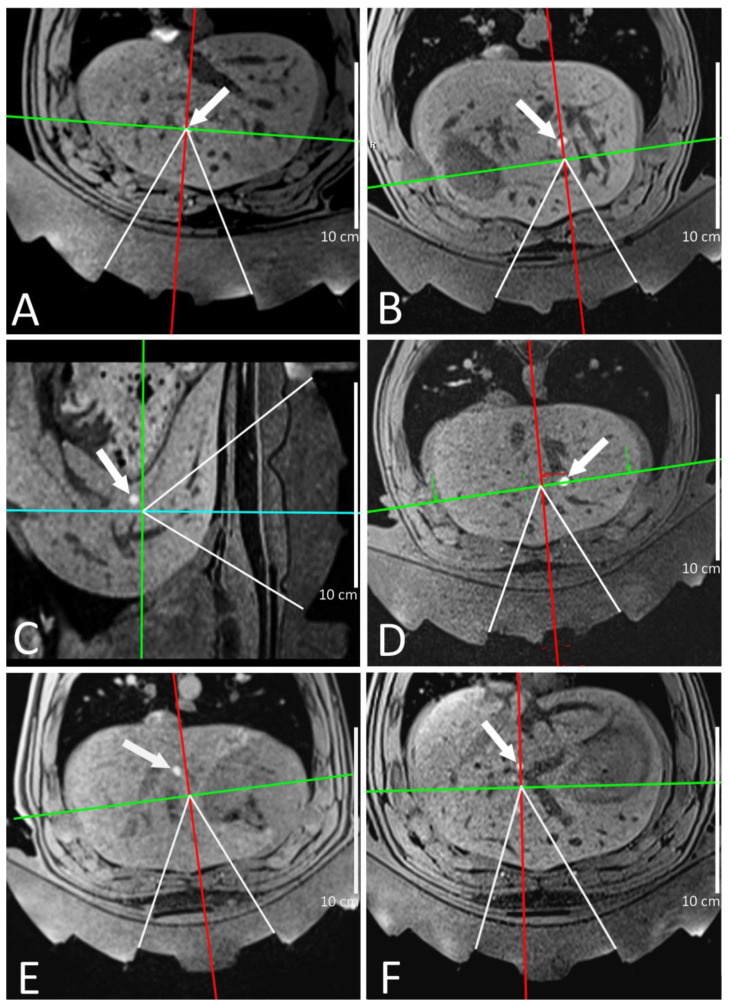
MR images of the MRgHIFU targeting in the 6 pig livers as follow: (**A**) pig 1, (**B**) pig 2, (**C**) pig 3, (**D**) pig 4, (**E**) pig 5, and (**F**) pig 5. The white arrow indicates the histological marker, visible as clear hypersignal on T1 sequences. The orthogonal lines indicate the planned MR thermometry slices that are crossing at the actual MRgHIFU target. The HIFU beam of the central segment of the transducer using electronic focusing per need (see Table 1) is indicated by the converging white lines.

HIFU targeting and ablation were guided by a 3T MR scanner (Prisma Fit, Siemens, Erlangen, Germany). A breath-hold 3D T1-weighted gradient-recalled echo MR sequence was acquired using two flexible body coils wrapping the animal, for targeting and dynamic contrast-enhanced (DCE) post-ablation imaging (see Figure 2). The following parameters were used: acquisition time (TA) = 54 s, repetition time (TR) = 4.08 ms, first and second echo times (TE1 and TE2) for Dixon reconstruction of 1.35 ms and 2.58 ms, respectively, flip angle (FA) = 9.5°, bandwidth (BW) = 1040 Hz/pixel, acquisition resolution = 0.8 × 1.1 × 1.4 mm^3^, 112 slices, reconstruction resolution = 0.8 × 0.8 × 1.2 mm^3^, slice oversampling 30%, no partial Fourier, parallel imaging GRAPPA = 2.

The natural focal spot location for a given orientation of the HIFU transducer was determined using the symmetry axes of that transducer and accurate measurements on the 3D high-resolution MR images, according to Lorton et al. [66]. The two-mirror symmetry planes of the applicator’s casting, reciprocally orthogonal, denoted as P1 and P2, were identified on the 3D T1w sequence, using the built-in software tools on the MR scanner (see Figure 1B,C). The intersection of P1 and P2 defined the main acoustic axis, where the natural focus should be found at a distance of 100 mm from the active surface.

The transducer was initially placed approximatively in front of the pig liver and adjusted based on the MR images. The actual target for MRgHIFU ablation was prescribed coplanar with the corresponding “pseudo tumor” histologic marker, either in the axial plane (5 pigs) or in the sagittal plane (1 pig, due to the presence of major blood vessels in the axial plane; see Figure 2). Except for pig 1, where the MRgHIFU target was the RF marker itself, we decided to distinguish the MRgHIFU target from the histologic marker by deliberate in-plane shifting (either axial or sagittal) in order to facilitate the post-treatment analysis. The targets were placed considering the local anatomic environment and steering capacities of the transducer. All targets were intra-hepatic, proximal to major blood vessels, and the target of pig 3 was also proximal to the stomach lining. The objective was to create MRgHIFU thermal ablations without inducing damage on tissues or organs at risk. The targeting images as shown in Figure 2 were recorded the day of intervention for comparison with the induced MRgHIFU ablations. The planned coordinates of the focal point, adjusted with electronic focusing per need, are provided in Table 1.

HIFU sonications were generated during breath-hold of duration ranging from 30 s to 40 s. Hepatic ablations were performed in 5–22 sonications using 700–1050 acoustic watts depending on the environment of the target and the tissue absorption, meaning a delivered energy ranging from 21 to 30 kJ per sonication.

The near real-time temperature monitoring during HIFU ablation was performed during breath-hold by PRFS MR thermometry using the following parameters: three orthogonal 2D slices acquired interleaved, with segmented echo planar imaging (EPI) factor = 7, temporal resolution = 2.7 s, field of view (FOV) = 256 mm, slice thickness = 4 mm, voxel size = 2.0 × 2.0 × 4.0 mm^3^, TR = 41.5 ms, TE = 10 ms, FA = 12°, BW = 814 Hz/pixel, and with spectral-selective fat suppression.

**Figure 3:**
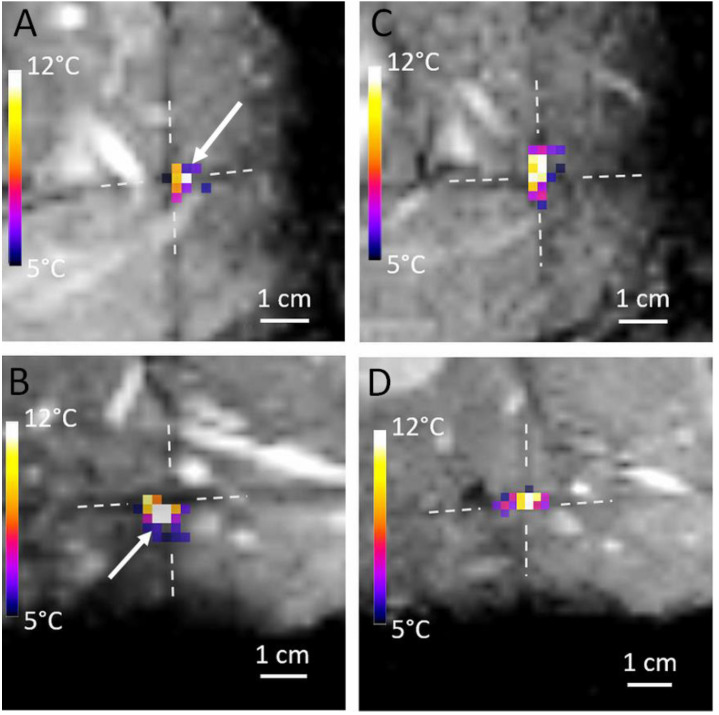
Temperature elevation maps at the end-point of sub-clinical sonication merged with MR magnitude images showing the iterative process to reach the target in the axial (**A**,**C**) and in the coronal (**B**,**D**) planes. The saturation of longitudinal magnetization from slice intersection yields hypointense bands, which are tagging the images. (**A**,**B**) the focal point near the intersection of three orthogonal slices, materializing the target, and (**C**,**D**) the adjusted focal point matching the respective intersection. The white arrows indicate the suboptimal location of the focal point.

Based on the 3D T1w MRI images, the unique point of intersection of the three MR thermometry slices was set on the acoustic target. The coronal slice was prescribed parallel to the HIFU applicator, while the axial and sagittal ones run alongside the acoustic axis. The prescribed slice geometry was exported from the 3D MPR card and loaded into the MR sequence-planning thread. We further exploited the negative contrast of the slice intersections in order to adjust and confirm the actual position of the HIFU focal point. Indeed, the saturation of the longitudinal relaxation of ^1^H spins, due to shorter effective TR, enables one to visualize the intersection of the planes as hypointense bands on MR thermometry magnitude images, and to visually attach this reference frame on the target coordinates initially defined on the 3D T1w MRI images. The focal point of sub-clinical sonications was iteratively displaced using electronic steering of the HIFU beam under MR thermometry monitoring, until it matched the unique point of three slices intersections, which corresponded to the expected target (see Figure 3).

The MR thermometry images were sent to the Thermoguide software v1.3.7 (Image Guided Therapy, Pessac, France) and merged with temperature maps in near real-time to monitor the thermal ablation and the eventual occurrence of ‘boiling core’ at the focus.

‘Boiling core’ would indicate that the tissues locally reach the threshold temperature near 100 °C and induces a state transition of the biological water from liquid to saturating vapor, along with the emergence of a bubble cloud [68]. It is identifiable as signal loss on T2*-weighted MR images and discontinuities in the complex phase signal caused by susceptibility artifacts.

Temperature maps merged with MR magnitude images were retrospectively analysed using Matlab R2016a (The MathWorks, Inc., Natick, MA, USA). A baseline correction of the temperature data was applied by subtracting the mean temperature calculated in a region of interest (ROI) in peri-vertebral muscle. The peak pixel was identified for each sonication as the greatest area under the temperature elevation curve, and served as a basis for estimation as to whether a lethal effect was reached. The plot of the average temperature elevation in a 4-pixel ROI around the peak voxel, as function of the time, was displayed for noise-attenuated visualization of the temperature trend.

### 2.3. Post-Treatment Follow-Up

A DCE MR sequence (described above) was performed at D7 post intervention on every animal (6/6). Immediately after animal sacrifice, necropsy was carried out by an expert abdominal surgeon to evaluate the HIFU effects on the tissues. Xypho-pubic abdominal laparotomy was performed and careful examination of the abdominal cavity was realized. The rib cage was also explored, looking for injuries resulting from the passage of HIFU energy.

A rating scale was defined to evaluate the near-field lesions induced by HIFU absorption in the ribs and sternum. The grades were numbered based on D7 post-intervention MRI and necropsy as indicated in Table 2. For each grade number, a sub-grade was added to distinguish between evidences on MR images or necropsy.

The liver specimen bearing the histologic marker and MRgHIFU ablation was isolated and fixated in formalin during 6 weeks. Post-fixation, the specimen was encapsulated in dual-component polyurethane foam. This step may be prone to uncontrolled specimen rotation during the bulk foam polymerization. Therefore, unlike Petrusca et al. [67], the encapsulated specimen was scanned with MRI to define the most appropriate orientation for further slicing. Subsequently, it was mechanically sliced 0.8 mm thick using a commercially available slicer (Gemma 300, SIRMAN s.p.a., Pieve di Curtarolo, Italy). Optical scans (600 or 1200 dpi) of each individual macroscopic slice were recorded. In contrast to Petrusca et al. [67], interslice co-registration was based on thin threads pre-printed in the foam material, perpendicular to the slicing plane. In-house-written Matlab code (The Mathworks Inc., Natick, MA, USA) was used for this purpose. Stacked tiff-type images were imported in OsiriX Software v13.0.1 (OsiriX Foundation, Geneva, Switzerland) for 3D post-processing.

The Gd contrast-enhanced MR images at D7 and the 3D reconstruction were compared side-by-side by a senior radiologist, and a radiological description of the lesions was compiled.

To determine the accuracy of the MRgHIFU lesioning, we measured the scalar distance between the center of the RF ablation and the center of the planned MRgHIFU ablation at D0. This metric was also determined at D7 on MR images and gross pathology, for comparison purposes in the same subject.

## 3. Results

**Figure 4:**
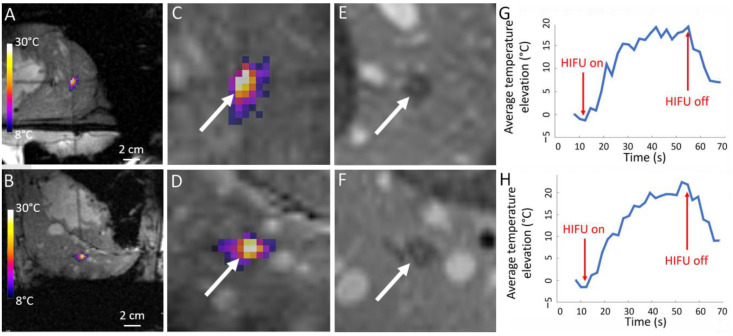
(**A**,**B**) Temperature elevation maps in pig 4 merged with MR magnitude images at the sonication end-point, sagittal and coronal. (**C**,**D**) Corresponding zoom-in on the focal spot indicated by the white arrow. (**E**,**F**) Gd contrast-enhanced MR images of the MRgHIFU ablation at D7 indicated by the white arrow. (**G**,**H**) Corresponding temperature elevation versus time in a 4-pixel ROI set on the focal point.

In all six pigs, the RF ablations were created to fixate the histological marker using 5–7 W during 10–16 s. The tumor-mimicking marker created by RF ablation and injection of Gd-chelate contrast agent was clearly visible on 3D T1-weighted MR images and allowed the MRgHIFU targeting as illustrated in Figure 2. The RF ablation sizes measured on D7 post-operatory Gd contrast-enhanced MRI are provided in Table 3, together with the metrics of the MRgHIFU ablation results. No complications or side effects due to the histological marker intervention, such as bleeding, hematoma or infection, were noticed in the six pigs. For each subject, the histological marker was eligible for MRgHIFU targeting and a thermal ablation by MRgHIFU was created in five of the six pigs. An illustration of the MRgHIFU lesioning process on pig 4 is shown in Figure 4. In one animal (pig 1), the lethal threshold of thermal dose was theoretically reached according to PRFS thermometry, but no ablation was visible on contrast-enhanced MR images after 7 days. The low number of sonications or an important blood flow could explain the absence of a permanent ablation. The liver of pig 1 will not be described in the following results as it was not analyzed on post-mortem examination due to the absence of radiological evidence on MR images at D7. Five liver specimens were isolated and fixated as described. Four out of the five liver specimens were successfully cut and digitized, while one liver specimen was lost from analysis due to technical issues.

**Figure 5:**
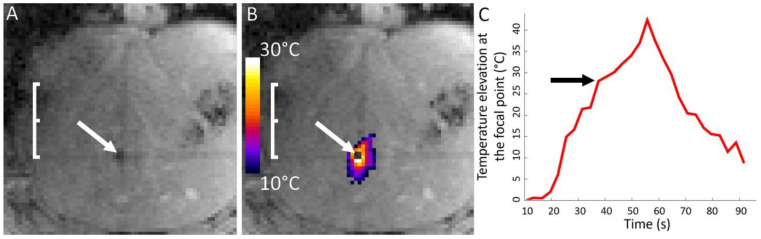
(**A**) Magnitude MR image of the boiling core. The white arrow indicates signal loss on T2*-weighted images. (**B**) Magnitude MR image merged with temperature elevation maps illustrating a ‘boiling core in pig 3′. The white arrow indicates a signal void in the temperature calculation due to signal loss magnitude images. (**C**) Corresponding temperature elevation in the warmest pixel. The black arrow indicates a slope discontinuity followed by supra-linear enhanced heating.

The maximal temperature elevation and the size of the histological marker and MRgHIFU ablations are presented in Table 3. In our study, three pig livers (2, 3, and 4) reached sufficient focal point temperature to induce ‘boiling core’ as illustrated in Figure 5.

**Figure 6:**
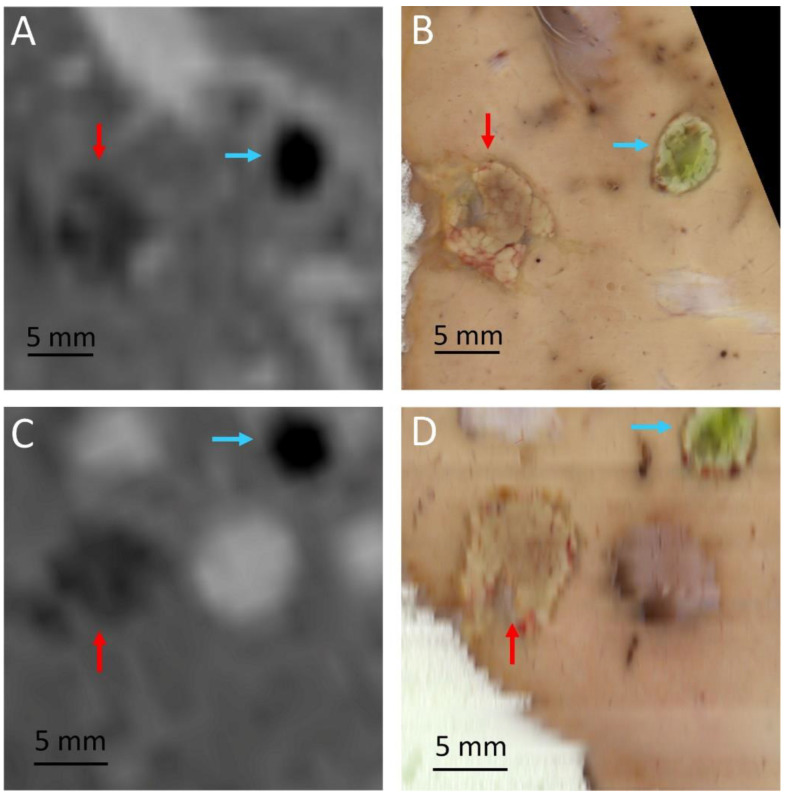
Post treatment follow up in pig 4, shown in two perpendicular planes from internal parenchyma, segment 4, close to the medium hepatic vein. Data were co-registered between MRI: (**A**,**C**), and gross pathology: (**B**,**D**). The red arrows indicate the HIFU ablation and the blue arrows the histologic marker (RF ablation).

The MRgHIFU ablations matched the expected locations within the millimeter accuracy range and were clearly visible post-operatory on DCE MRI at D0 and D7. The RF ablation yielded a deep signal void on DCE MRI and its shape was ellipsoidal (see Figure 6A,C). The MRgHIFU ablation is less hypointense on DCE MRI and less regularly shaped. According to two expert abdominal radiologists, differentiating the two ablation entities was beyond doubt. The histologic marker was readily identified on gross pathology due to methylene blue staining.

**Figure 7:**
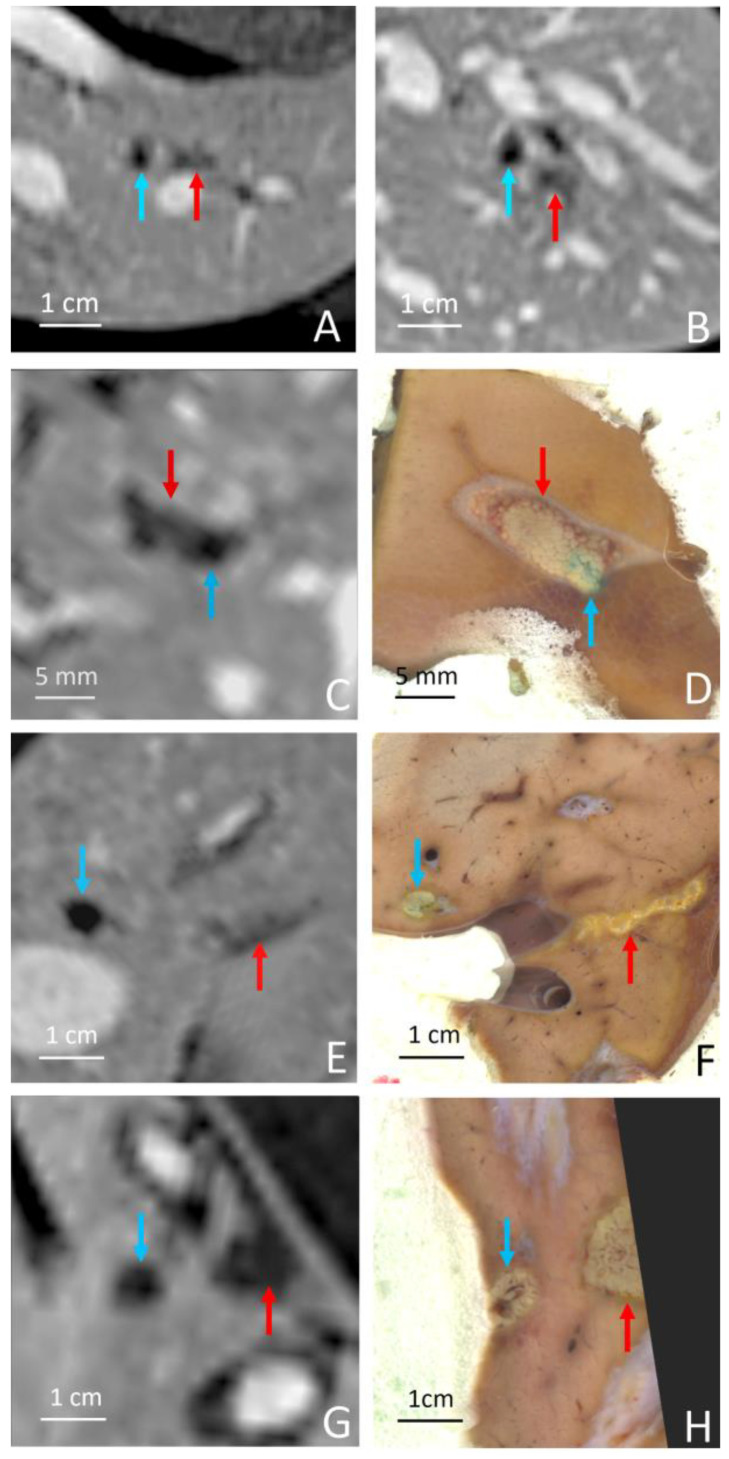
Coplanar images of the histologic marker (blue arrow) and the HIFU ablation (red arrow) for pig 2 (**A**,**B**), pig 3, (**C**,**D**), pig 5 (**E**,**F**) and pig 6 (**G**,**H**). Gd-enhanced MRI and gross pathology are shown as available. The MRgHIFU ablations are located in (**A**,**B**) left medial hepatic vein, segment 4 (**C**,**D**) deep parenchyma, segment 4, close to the bifurcation with the portal vein; (**E**,**F**) close to the left hepatic vein, and (**G**,**H**) left liver, segment 3, close to the left portal vein.

The graph of the temperature elevation versus time indicated a slope discontinuity as well as supra-linear enhanced heating, after the onset of the bubble cloud. The shape of the MRgHIFU ablation was found to be similar between post-operatory MRI and gross pathology in every analyzed case; see for illustration Figure 6 and Figure 7.

The focal-point-to-rib heating contrast, defined as their temperature elevation ratio for a given sonication, was found to be 7.3 (1.8–17.0). However, both temperatures were recorded only in the monitored slice in a 4-pixel diameter region of interest around the maximum temperature.

Gross pathology after formalin fixation confirmed the visual differentiation of RF ablation and MRgHIFU ablation, the RF ablation being colorized by methylene blue (see Figure 6B,D and Figure 7B,D,F,H). As illustrated in Figure 6 and Figure 7, the delineation between the MRgHIFU ablation and the healthy tissues is clear and submillimetric.

**Figure 8:**
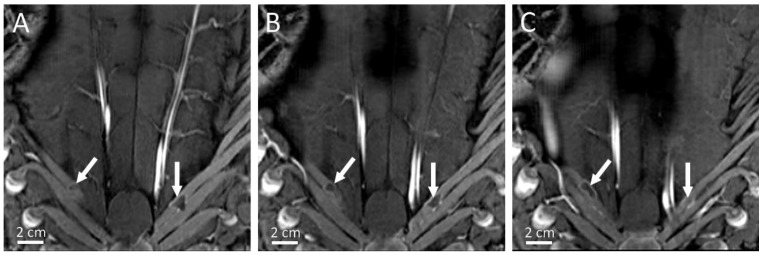
Curved MR image reconstruction of the rib cage of pig 5 illustrating thermal lesions visible at D7 on Gd-enhanced 3D T1w MRI in three adjacent slices of 2 mm thickness (**A**–**C**). The grade 1a thermal lesions are highlighted by the white arrows.

During post mortem examination, no skin lesion was detected on the thoracic and abdominal regions. Ribs were meticulously dissected and one thermal lesion of 2 mm length on the side exposed to HIFU was visually detected. Therefore, one pig was assigned a grade 1b rib lesion. Moreover, a few millimeter-sized rib thermal lesions of grade 1a were assigned in five of the six pigs during the MRgHIFU interventions, without any evidence found during post-mortem examination (see Figure 8). Two pigs had a sternum thermal lesion; one grade 1a was due to the absence of an acoustic absorbent patch, and a grade 1b lesion was due to the wrong location of the patch. After the incident, the absorbent patch was placed based on pre-interventions anatomical MR images. The completion of the thoracotomy and the exploration of the internal face of the rib cage revealed no more evidence that HIFU adverse side effects occurred.

No superficial lesions involving the skin, the subcutaneous tissue, nor the muscle wall have been evidenced. Likewise, visual inspection of the whole peritoneal cavity did not reveal any lesions on the internal face of the abdominal wall, nor on the organs that had not been targeted by MRgHIFU.

On exploration of the liver, the RF ablations were easily detectable. At the defined target site, MRgHIFU treatment induced easily identifiable ablations. These ablations were palpable and easily recognizable as a harder tissular nodule compared with surrounding healthy (untreated) liver parenchyma. Moreover, in the case of a subcapsular, superficial lesion, an evident promptly detectable, whitish lesion was localized. No adherence was detected between the hepatic lobes, and surgical manipulation of the liver itself was also found to be standard. The whole liver was harvested and ex situ sliced to find the MRgHIFU ablation(s).

As indicated in Table 3, the dimensions of MRgHIFU ablations found on gross pathology were in agreement with DCE MRI measurements. Furthermore, the scalar distance between the marker and the MRgHIFU ablation was in good agreement with the planned geometry. The reported accuracy of 2.4 ± 2.0 mm is suitable for hepatic interventions.

## 4. Discussion

In a previous report [66], the performance and workflow of the current MRgHIFU prototype device were demonstrated in hepatic tissues without considering the local anatomy, nor ablating a priori defined targets in challenging locations. In the current report, we addressed the question of focusing the HIFU beam on a pre-existing tumor-mimicking marker. This setup questioned (1) the ability of this device to be steadily positioned in front of the abdomen, (2) the ability of our software tools to plan and execute targeted ablations, and (3) the ability of the investigators to proceed to intra-operatory fine adjustments of the focal point position in a complex biological tissue, under visual guidance by real-time MR thermometry, converging on the tagged target in a reasonable duration.

The intrinsic acoustic absorption in the bone (e.g., rib cage) is approximately 12 times [69] higher than in soft tissue. This condition yields a severe risk for adverse side effects on the rib cage for transcostal sonication. The central goal of our technology is to effectively lower the ratio of the temperature elevation on the ribs versus the focal point, reaching a safety level where no specific means of protection for ribs are required during the sonication. Inspired by the Fresnel’s lens principle, our multi-segment confocal design offers natural “super-focusing” in deep tissue, large bend radius and hence optimal abdominal fitting, and wide aperture, yielding two orders of magnitude lower acoustic intensity at the location of the ribs as compared with the focal point.

Our results suggest this novel phased-array super-convergent transducer allows targeting of deep intrahepatic tumors and minimizes the impact on the rib cage exposed to HIFU. The results also suggest that this device does not require specific precautions for ribs as the eventual thermal lesions on ribs were millimeter-sized, and of grade 1a (*n* = 5) and grade 1b (*n* = 1). Such minimal rib lesions due to HIFU exposure do not significantly alter the benefit/risk ratio in the life-threatening oncologic context of liver malignancies as compared with the infiltrating and necrotic lesions in the near field that may occur. In addition, as illustrated in Figure 8, the therapeutic ultrasound propagation may be more difficult in pigs than in humans, as the inter-costal spaces are tighter in pigs. The ratio between the temperature elevation at the focal point versus the temperature elevation in the ribs was, on average, 7.3, as measured with MR thermometry in a single axial slice available here. This result further supports the relevance of the tailored transducer design to minimize near-field side effects. Three-dimensional volumetric monitoring of the rib cage heating was desirable but not available. Avoiding the need for rib protection largely facilitates the workflow. It also eliminates the risk of displacement of such means of protection during the intervention. Nevertheless, MRgHIFU ablation necessitates a protective layer for the sternum that is central, symmetric and easier to identify and shield than ribs.

Until now, the application of MRgHIFU to liver thermal therapy was very sparse, with only a few case reports available in the literature. Okada et al. [52] reported a case of hepatocellular carcinoma treated by MRgHIFU in a conscious patient in 2006. A few cases of MRgHIFU treatment in the low liver or the left lobe through the epigastrium under general anesthesia and controlled breathing were reported by Gedroyc [70] and Fisher et al. [71]. Anzidei et al. reported in 2014 a series of seven hepatic and two pancreatic procedures [46,72] under general anesthesia in carefully chosen patients to prevent near-field obstructions, all in easily accessible areas of the organ. Our in vivo results suggest the design and performance of this novel concept of MRgHIFU applicator may break a technology lock, in order to translate into clinical practice the theoretical potential of this approach and fulfil its promises.

Ultrasound propagation in complex biological tissue is subjected to reflection, refraction and the production of stationary waves. Electronic focusing using the theoretical time-of-flight in a homogeneous medium is suboptimal. Intra-operatory sub-clinical iterative sonications are requested for visual adjustment of the actual focal spot position. Here, a T1-based saturation mechanism of the MR signal at slice intersection was exploited for “tagging” the target on PRFS MR thermometry data.

Furthermore, significant acoustic energy travels behind the HIFU focal point [68]. Undesired post-focal thermal lesions are possible at interfaces, e.g., soft tissue-air or soft tissue-bone, even when the energy deposition at the focal point is sub-lethal. In this study, high-resolution contrast-enhanced MRI as well as visual examination by an expert abdominal surgeon during necropsy excluded post-focal thermal lesions.

The volume of the MRgHIFU ablations reported in this study is too low to be used as clinical routine for complete ablation of hepatic malignancies. However, the occurrence of boiling core, which was observed in our study, may offer the possibility of creating larger ablations in future studies. It acts as a reflector for acoustic waves at the bubble cloud interface, enhancing the energy deposition in the inferior part of the focal area by addition of the propagating and the reflected waves. On the other hand, the bubble interface operates as a safety barrier for the far field, protecting the healthy tissues from heating. This has been previously described in ex vivo organs [68,73], but to our knowledge, this is the first in vivo demonstration under MRI monitoring.

On the other hand, infra-centimeter-sized ablations are of great interest in a tumor-tagging protocol, namely ‘tumor tattooing’, pointing out the region scheduled for resection. Visualization on pre-operatory MR images and intra-operatory palpation of adjuvant MRgHIFU ablations may improve the patient outcome amenable for surgical resection of malignant tissues. ‘Tattooing’ before chemotherapy could also highlight the locations of malignant tumors for cancer follow-up, which may macroscopically disappear after chemotherapy. Our technology seems suitable to sustain this promising adjuvant technique to help accurate parenchyma resection.

The RF marker was placed in a location reachable by the MRgHIFU transducer between 80–120 mm from its active surface. The electronic beam formation and the design of the device may limit the volume reachable by ultrasound and restrict the number of eligible patients. To overcome this issue, one solution may be the geometric scaling of transducers into a set of several sizes and choosing the appropriate version on a case-specific basis.

## 5. Conclusions

The described device and workflow were suitable for transcostal hepatic ablations in pig liver, targeting pre-defined locations. Efficient focusing yielded 58–86 °C on deep-seated targets. MRgHIFU ablations were induced in challenging sites for resection, with high targeting accuracy (2.4 ± 2.0 mm). Specific means of protection for the ribs are not required for this intervention but a reflective layer is needed for sternum protection.

## Figures and Tables

**Table 1:** Coordinates of the focal point in the reference system of the natural focal point.

Pig	Focal Point Coordinates along the Short Axis, Long Axis and Acoustic Axis of the Transducer [mm]
1	(0, 10, 2)
2	(0, −11, 7)
3	(2, 0, 7)
4	(0, 0, 1)
5	(0, 0, 0)
6	(0, −15, −7)

**Table 2:** Rating scale for the near-field thermal lesions in pigs at D7 post-intervention.

Grade	Evidence	Sub-Grade
0	No evidence of near-field thermal lesion	
1	Thermal lesion on the anterior surface of the bone facing the HIFU transducer	a: DCE MRI only b: necropsy-based visual evidence
2	Thermal lesion on the entire circumference of the bone	a: DCE MRI only b: necropsy-based visual evidence
3	Thermal lesion in the surrounding soft tissues juxtaposing the anterior surface of the bone facing the HIFU transducer	a: DCE MRI only b: necropsy-based visual evidence
4	Thermal lesion in the surrounding soft tissues anterior and posterior to the bone	a: DCE MRI only b: necropsy-based visual evidence

**Table 3:** Overview of the thermal ablations in the pig livers.

Pig	RF Ablation Size (mm^3^), from MR Images (along AP, LR and HF Axes)	Temperature Reached During HIFU Ablation (°C)	HIFU Ablation Longest Axis (mm) from MR Images; See Figure 6 and Figure 7	HIFU Ablation Longest Axis (mm), from Gross Pathology; See Figure 6 and Figure 7	Planned Center-to-Center Distance between the RF and MRgHIFU Ablations (mm), from MR Images	Center-to-Center Distance between the RF and MRgHIFU Ablations (mm), from Gross Pathology	Center-to-Center Distance between the RF and MRgHIFU Ablations (mm), from MR Images	Near-Field Side Effects Grade
1	4.9 × 6.1 × 4.8	63	-	-	0	-	-	1a
2	7.2 × 6.3 × 9.2	86	6.9	-	11.8	-	10.1	1a
3	4.6 × 3.7 × 4.5	85	15.8	16.5	6.7	3.6	3.2	1a
4	6.7 × 6.1 × 6.8	86	7.4	7.9	16.1	16.0	16.2	1a
5	5.1 × 4.7 × 6.2	62	21.1	23.2	18.4	24.2	24.1	1a
6	3.0 × 4.6 × 5.5	58	14.0	15.0	11.2	10.3	10.3	1b

## Data Availability

All data are available upon request.
